# Against Cursory Treatments in Ethics of Medical Migration from Underserved Countries

**DOI:** 10.1007/s11673-017-9782-0

**Published:** 2017-03-09

**Authors:** Yusuf Yuksekdag

**Affiliations:** Centre for Applied Ethics, Linköping University, Hus Key, Campus Valla, 58431 Linköping, Sweden

**Keywords:** Medical migration, Ethics, Right to exit, Retention

## Abstract

In a recent paper, Mpofu, Sen Gupta, and Hays (2016) attempt to outline the obligations of recruiting high-income countries (HICs) and would-be emigrant health workers (HWs) to tackle the effects of mass exodus of health workers from underserved regions. They reconstruct (i) Rawlsian and Kantian global justice approaches to argue for moral obligations of HICs and (ii) an individual justice approach to point to non-enforceable social responsibilities of HWs to assist their compatriots. This critical commentary demonstrates that the argumentation within their individual justice approach is problematic on the basis of three reasons: (1) their discussion under-theorizes and undervalues individual rights and more specifically the right to exit, (2) their argumentation in the latter part, even if problematically, does rather point to moral obligations in lieu of social responsibilities of HWs, and (3) they overlook many other important freedoms, interests, and values pertinent to the issue of retention.

In their article on ethics of medical migration from underserved regions, Mpofu, Sen Gupta, and Hays (2016) attempt to outline moral obligations of recruiting high-income countries (HICs) and social responsibilities of would-be emigrant health workers^1^ (HWs) to tackle the diminishing effects of mass exodus of health workers on health delivery systems in underserved regions. First, they reconstruct Rawlsian and Kantian approaches to global justice and argue for moral and corrective obligations of HICs to ensure global health equity to some extent. Second, they provide an individual justice approach to point to non-enforceable obligations (“social responsibilities” in their terminology) of HWs to assist their compatriots that in the end translates into a formal ethical-reflection training for medical students to consider retention (Mpofu, Sen Gupta, and Hays 2016, 401). While there is much to be praised in their multi-layered attempt to provide concrete and ethically reflected policy suggestions, the authors, nevertheless, have a quite cursory treatment of certain components of their individual justice approach, and the article also fails in accommodating many other important considerations pertinent to retention in underserved regions. This critical commentary has three claims:

1. Their discussion on balancing (a) the value of the right to exit with (b) the interests of vulnerable populations under-theorizes individual rights and undervalues what the right to exit entails;
2. Their methodology rather points to moral obligations in lieu of non-enforceable social responsibilities for HWs;
3. Their discussion of individual freedoms misses many other important freedoms, interest, and values pertinent to the issue of retention in underserved areas.

Within their individual justice approach which is claimed to be based on individual rights and social responsibilities, the authors try to account for why HWs would have social responsibilities to assist vulnerable populations. They conceptualize their understanding of social responsibilities in the following way:

Social responsibility, whether enacted by an organization or an individual, is the imperative, without compulsion from an external sanction or authority, to make decisions on the basis of that which will do the most for society at large, even if that means sacrificing the personal wants and/or needs of the decision-maker. (Mpofu, Sen Gupta, and Hays 2016, 401).

From this point onward, the authors principally aim at providing the reasons why HWs should ethically reflect and curtail (a) their aspiration to migrate, in the face of (b) the healthcare needs of vulnerable populations. The authors start their own reflection by discussing the value of the right to exit and by providing valid reasons for its constraints and limitations.

(1) This reflection, firstly, under-theorizes individual rights. After listing the arguments in favour of recognizing the right to exit as well as citing Article 13 of the Universal Declaration of Human Rights (UDHR) on the right to emigrate, the authors claim that this does not entitle any individual to the right to hold a particular profession, in this case a medical post, in one of the HICs. This is not a controversial claim. The right to exit alone would not vindicate a right to be accepted to positions available in HICs. It would also be counterintuitive to argue that the states concerned have a duty to provide jobs for the HWs or any other individual. However, it seems there is a misreading regarding the notion of rights here. The authors conclude that this, presumably, implies that HICs do not have a duty to allow HWs to apply for medical posts within their territory (Mpofu et al. 2016, 402). This conclusion does not follow from their premise. It would, in fact, be the duty of HICs not to prevent HWs, or any other individual for that matter, from applying for such jobs. There is a certain confusion and undertheorizing regarding what rights and their correlative duties imply for the corresponding institutions (Feinberg 1966).

Another example is the authors’ use of the Kantian imperative of respecting individuals as an end in themselves to contend for a certain constraint on the right to exit of a health worker. They claim that an emigrant health worker can also be considered to be using the population as a means to her ends. The reason is that, if trained with the public funds, an emigrant health worker would be using the benefits of the publicly contributed funds to advance her personal interests (Mpofu et al. 2016, 402). Putting this unusual reading of Kant aside, it is not clear why this line of thinking implies that a health worker should consider retention, rather than paying back her fair share in the form of an exit tax (Brock and Blake 2015). Placing obligations on a skilled labourer, nonetheless, is a more vivid case of using an individual as a means to an end, as it implies using one’s skills and talents to tackle the diminishing healthcare delivery in a region.

Secondly, the discussion undervalues the right to exit. Mpofu and colleagues’ cursory and instrumentalist treatment leaves the right in question without much theorizing regarding its grounds. They seem to take an individual’s exercise of the right to exit as a means to “further one’s desires” or get “higher salaries” (Mpofu, Sen Gupta, and Hays 2016, 402). The right to exit, nonetheless, can be valued on many different grounds rather than simply increasing the amount of life-options a person can enjoy (Oberman 2016). The right to exit can be considered integral to protecting bodily and material safety of an individual, or providing a voice to ensure a certain level of political accountability, or even as a fundamental right to protect one’s basic normative agency at the international level^2^ (Lenard 2015; Cole 2012). There are considerable numbers of emigrant medical graduates from underserved regions such as Sub-Saharan Africa, who migrate abroad for reasons such as education, political instability in their home country, or family reunification (Poppe et al. 2016). The authors’ cursory treatment of the right to exit arises mainly because they primarily focus on the valid reasons why the right to exit should be constrained. This brings us to my second claim.

(2) The authors discuss the valid reasons for constraining the right exit in order to argue for a non-enforceable obligation of HWs to assist their compatriots. There is a methodological confusion with this line of reasoning as it implies more than what is intended. Given that the authors conclude with an argument for ethical-training to promote awareness to achieve retention, it is not clear why the authors methodologically dissect the arguments in favour of constraining the right to exit.^3^ The reasons why a health worker should choose to stay is not a question of if she should or should not be entitled to the right to exit or whether or not there are justifiable constraints on the right. Their rights talk, even if it has issues for the reasons discussed in (1), rather points to a call for specification of the right on the level of political policymaking. If specified on the basis of valid reasons, the states then would hold the prerogative to prevent or delay the exercise of the right in question. I should note here that it is one thing to argue for moral obligations of health workers to assist their compatriots and another thing to contend that this implies an unconditional duty to stay as the latter should account for the burdens they will face under the given conditions. However, the authors discard this possibility altogether as they confine their responsibility model into non-enforceable obligations. This is also surprising considering their call for moral obligations of HICs towards low- and middle-income countries (LMICs) on the basis of the inefficiency of non-coercive imperatives such as ethical recruitment (Mpofu, Sen Gupta, and Hays 2016, 400).

(3) A possible, and more generous, reading of the article might suggest that individual rights approach is rather an analytical starting point to tease out the interests of HWs in migrating abroad. While this is worthwhile to pursue, the project, nevertheless, suffers from two problems. The first is the abovementioned undervaluing problem. To do justice to any form of reflection on individual interests and potential social responsibilities to achieve non-enforced retention, the authors’ account should pay attention to what the fundamental interests behind the right to exit entails more conscientiously.

The second issue is the scope of the authors’ approach. Mpofu and colleagues delimit their discussion on the interests of HWs to a discussion on the right to exit. This is not a fruitful choice, as they miss many significant rights, interests, and considerations which are relevant to retention in LMICs. The reasons why a health worker should consider retention has little to do with the values behind the right to exit, since most of the worry is embedded not in the initial choice but rather within the duration of their stay. The real ethical and empirical challenge lies in providing a framework which also discusses the interests of HWs in free occupational choices, fair contracts, well-functioning working conditions, and viability and efficiency of retention in LMICs. The right to exit, as an analytical starting point, does not grasp all the values behind these interests and concerns. Take the example of medical graduates who aspire to migrate abroad to pursue a different profession. The right to free choice of employment is already recognized under the Article 23 of UDHR. It is not clear, in the paper, if the medical graduates who wish to pursue other professions abroad would have a social responsibility to stay and assist their compatriots. This would point toward a very "directive" ethical reflection which warrants a closer look on its own (Owen 2016, 63).

The aim of the individual justice approach in the article is simply to provide a sound basis for the curriculum of the ethical reflection training of HWs in LMICs. This is an insightful approach to the issue of retention, yet an underworked one. It is not obvious, in the end, if the approach does offer adequate guidance for such a curriculum.

## Footnotes

1 The authors do only refer to social responsibilities of medical practitioners in the article, while their main concerns and analyses are pertinent to health workers in general.

2 Examples would be, respectively, exercising territorial exit to escape oppression, how being entitled to territorial or associational exit would create another form of ensuring accountability in policymaking, and individuals being primarily international agents caught up in global labour demands within our contemporary world (Lenard 2015; Cole 2012).

3 This is not to say that rights, or the right to exit in this case, cannot be delayed or constrained to balance it with more fundamental rights or interests, as in the case of the effects of medical practitioner migration from underserved regions.
